# Comparative Evaluation of Metabolic Syndrome, Circadian Syndrome, and Allostatic Load Measures on All‐Cause Mortality in U.S. Males and Females

**DOI:** 10.1155/ije/7064400

**Published:** 2026-05-11

**Authors:** Shakeel Ahmed, Pallavi Dubey, Randi E. Foraker, Alok Kumar Dwivedi

**Affiliations:** ^1^ Department of Molecular and Translational Sciences, Paul L. Foster School of Medicine, Texas Tech University Health Sciences Center, El Paso, Texas, USA, ttuhsc.edu; ^2^ Department of Obstetrics and Gynecology, Paul L. Foster School of Medicine, Texas Tech University Health Sciences Center, El Paso, Texas, USA, ttuhsc.edu; ^3^ Department of Biomedical Informatics, Biostatistics and Medical Epidemiology, Center for Integrated Biostatistics and Epidemiology, University of Missouri School of Medicine, Columbia, Missouri, USA, missouri.edu

**Keywords:** all-cause mortality, allostatic load, circadian syndrome, metabolic syndrome, stratified Cox regression

## Abstract

**Background:**

Metabolic stressors, including metabolic syndrome (MetS), circadian syndrome (CircS), and allostatic load (AL), measure the metabolic and stress‐related physiological dysregulations that are associated with the risk of frailty and cardiovascular mortality in the adult population.

**Aim:**

To assess the effects of MetS, CircS, and AL on all‐cause mortality in U.S. adults by age and sex.

**Methods:**

We performed a retrospective cohort analysis using the National Health and Nutrition Examination Survey (NHANES) data, including 7 cycles (2005–2018). The associations between metabolic stressors and mortality were evaluated using stratified Cox regression analyses.

**Results:**

The study included 37434 adults for analysis of CircS and MetS, while 26480 adults were included for AL analysis. All exposures, including CircS (hazard ratio [HR]: 1.29; 95% confidence interval [CI] 1.18–1.42), MetS (HR: 1.09; 95% CI 1.00–1.18), and AL (HR: 1.25; 95% CI 1.11–1.41), were associated with all‐cause mortality in the entire cohort. In adjusted analyses, CircS (HR: 1.55; 95% CI 1.34–1.80), MetS (HR: 1.32; 95% CI 1.14–1.53), and AL (HR: 1.44; 95% CI 1.21–1.70) were only associated with all‐cause mortality in individuals with age < 70 years. In subgroup analyses by sex, all three exposures were strongly associated with mortality in males as well as in the subgroup of males with age < 70 years. The CircS was found to be a strong marker for all‐cause mortality in individuals aged < 70 and in both sexes.

**Conclusion:**

The strength of association between CircS and all‐cause mortality was stronger than that of MetS and AL, irrespective of sex. Our findings indicate that metabolic biomarkers, specifically CircS, may help refine risk stratification and prognostic evaluation among adults aged less than 70 years.

## 1. Introduction

Metabolic stressors are common abnormalities affecting over 30% to 40% of individuals over the life course [1]. Metabolic stressors contribute to multiple metabolic diseases, including diabetes, metabolism‐associated fatty liver disease, cardiovascular disease (CVD), and different cancers. Metabolic syndrome (MetS) is a well‐known public health crisis that continues to plague healthcare systems globally. While the overall prevalence of MetS in the United States (U.S.) has remained stable, a significant increase has been noted among young adults and between races/ethnicities from 2011–2012 to 2015–2016 [2]. MetS is known to be strongly associated with an increased risk for chronic conditions and early mortality from CVD, yet there is a lack of consensus on specific factors influencing these outcomes [3, 4]. As CVD remains the leading cause of mortality globally [5, 6], identifying additional preventive factors is critically needed to minimize the risk of CVD and associated poor health outcomes. There is emerging evidence that additional markers of metabolic stressors, such as circadian syndrome (CircS) and allostatic load (AL), may be beneficial for CVD risk stratification [7–10].

The CircS plays a crucial role in regulating human health and metabolism. Most cells and tissues operate on a 24‐h rhythm that contributes to well‐being and homeostasis [11]. This rhythm is controlled by specific genes and their protein transcription factors that regulate the expression of other genes, creating a feedback loop to maintain the rhythm at the molecular level [12, 13]. Dysfunction in this rhythm, known as CircS, is associated with changes in sleep/wake patterns, eating habits, and diet, which increase the risk of chronic diseases, such as CVD [13, 14]. These factors are prevalent in the community due to the impact of modern society, including increased shift work, light exposure, sleep disorders, and changes in nutrition [13, 15, 16]. CircS has been associated with increased comorbidities that coincide with the MetS, with the presumption that there is a common etiology [7]. In recent studies, CircS was not only associated with CVD but also found to be a better predictor when compared to MetS alone for CVD risk [10, 17]. However, no significant differences between CircS and MetS were found in relation to CVD mortality [11].

AL, a measure of cumulative physiological stress, may increase the risk of CVD and all‐cause mortality [18]. Previous studies have shown that markers of AL increase the risk of CVD, cognitive decline, and all‐cause mortality due to the body’s pathophysiological response [19]. AL is mediated by glucocorticoids, dehydroepiandrosterone (DHEA), cytokines, and catecholamines over time as they help the body respond to stress [9]. These metabolic stressors are known to be preventable through early screening and therapeutic management, resulting in reduced risk of CVD and associated adverse health outcomes. Despite their usefulness, these markers, specifically AL and CircS, are rarely examined in routine physical or clinical evaluation. The relative performance of these markers on all‐cause mortality has not yet been evaluated. Furthermore, these measures might differ in terms of age and sex, as the differential prevalence of these markers is evident by age and sex [20–22]. We sought to evaluate the relative performance of measures of metabolic health stressors, especially MetS, CircS, and AL, on all‐cause mortality by age and sex. Although these measures capture different dimensions of metabolic/physiological stress and differ in conceptual basis and component structure, they share some markers of metabolic/physiological stress, precluding a direct comparison of these constructs in statistical modeling. Given this design, our aim was to estimate and compare the magnitudes and consistency of associations and discrimination metrics across construct‐specific models, rather than to obtain a direct head‐to‐head effect estimate under a unified model.

## 2. Materials and Methods

### 2.1. Study Design

A retrospective cohort study was conducted using the National Health and Nutrition Examination Survey (NHANES) by linking baseline data to all‐cause mortality from 2005 to 2018. The NHANES, conducted by the Centers for Disease Control and Prevention, is a national‐level study that updates data on the health and nutritional status of the U.S. population [23]. The NHANES collects data from U.S. participants through questionnaires, interviews (both telephone and in‐person), physical examinations, and laboratory tests. Interviews typically take place in participants’ homes, while blood sample collections and other physical examinations [24] are performed at mobile screening centers.

### 2.2. Data Extraction

Our study incorporates seven consecutive survey cycles of NHANES from 2005 to 2018, including 2005‐06, 2007‐08, 2009‐10, 2011‐12, 2013‐14, 2015‐16, and 2017‐18. The AL markers were only available for the cycles 2005–10 and 2015–18, and thus, only a subset of the study samples was included for AL‐related analysis. Missing data on mortality, MetS, CircS, AL, and pregnant women were excluded from the study. A flowchart for data inclusion in the final analysis is presented in Figure S1.

### 2.3. MetS

MetS was defined by the presence of three or more factors out of five components: (i) elevated triglycerides (≥ 150 mg/dL) or use of lipid‐lowering medications; (ii) waist circumference ≥ 88 cm in women and ≥ 102 cm in men; (iii) high blood pressure (systolic blood pressure ≥ 130 mm Hg or diastolic blood pressure ≥ 85 mm Hg) or the usage of antihypertensive medications; (iv) decreased high‐density lipoprotein (HDL) (< 40 mg/dL in men and < 50 mg/dL in women) or the use of lipid‐lowering medications; and (v) increased fasting glucose in blood (≥ 100 mg/dL) or the use of antidiabetic medications [25]. The cases with missing values in more than three components were considered missing in MetS and excluded from the study.

### 2.4. CircS

The CircS was defined by the presence of four or more factors out of seven components: (i) elevated triglycerides (≥ 150 mg/dL) or use of lipid‐lowering medications; (ii) waist circumference ≥ 88 cm in women and ≥ 102 cm in men; (iii) high blood pressure (systolic blood pressure ≥ 130 mm Hg or diastolic blood pressure ≥ 85 mm Hg) or the usage of antihypertensive medications; (iv) decreased HDL (< 40 mg/dL in men and < 50 mg/dL in women) or the use of lipid‐lowering medications; (v) increased fasting glucose in the blood (≥ 100 mg/dL) or the use of antidiabetic medications; (vi) declined sleep duration (≤ 6 h per day), and (vii) depressive symptoms as defined by the Patient Health Questionnaire‐9 (PHQ‐9) with PHQ‐9 ≥ 5 considered depression [17]. The cases with missing values in more than four components were defined as missing CircS and were excluded from the analysis.

### 2.5. AL

AL was constructed from 8 biomarkers, i.e., (i) systolic blood pressure, (ii) diastolic blood pressure, (iii) 60‐s pulse count, (iv) C‐reactive protein, (v) HDL, (vi) total cholesterol, (vii) creatinine clearance, and (viii) serum albumin, that show dysregulation of physiological systems used to protect the individual from disease risk [26]. A high threshold for each biomarker was determined based on clinical recommendations for health risk [8, 20]. For high AL, a high threshold below the 25^th^ percentile was defined for albumin and HDL, while a threshold set above the 75th percentile was used for the rest of the biomarkers. Diastolic and systolic blood pressures were computed as the average of 4 readings taken by trained medical personnel. The total AL score was obtained after summing the points from the 8 biomarkers, with a total load score of 8. A higher AL score is considered an indication of poorer health [27]. A total AL score of 4 or above is considered a high AL based on previous studies [28]. The cases with missing values in more than four components were declared missing AL and were excluded from the analysis.

### 2.6. Definition of All Exposures

MetS exposure was defined as the presence of any three of five conditions: elevated triglycerides, high blood pressure, high fasting glucose, decreased HDL, and increased waist circumference. CircS was defined as the presence of any four of seven conditions: elevated triglycerides, high blood pressure, high fasting glucose, decreased HDL, increased waist circumference, decreased sleep duration, and increased depression scores. AL was defined as the presence of any four out of eight conditions: high systolic blood pressure, low diastolic blood pressure, decreased HDL, high total cholesterol, increased pulse count, high C‐reactive protein, high creatinine clearance, and low albumin concentrations. Compared to AL and MetS, which are based on blood markers, CircS is partially based on self‐reported depression scores and sleep duration, which may introduce measurement error and recall bias.

### 2.7. All‐Cause Mortality

The mortality data were obtained from the public‐use versions of the linked mortality follow‐up files, which consisted of follow‐up mortality data from the survey participation date through December 31, 2019 [29]. The mortality status of each participant in the NHANES was obtained from the death certificates using the National Death Index (NDI) and linked with probabilistic matching methods. The follow‐up time for mortality status was derived from the date of the survey to the year of death or the end of the mortality follow‐up date, i.e., December 31, 2019.

### 2.8. Covariates

To adjust the effects of covariates on mortality, we included age (years), marital status (married, other categories, and unknown), sex (male vs. female), education level (lower than high school, high school diploma or general equivalency diploma, high school diploma and above qualification, and unknown), ethnicity (Hispanic, non‐Hispanic, non‐Hispanic black, and other racial groups), smoking status (smoker, nonsmoker, and unknown), alcohol usage (yes, no, and missing), and physical activity (yes, no according to the Global Physical Activity Questionnaire [GPAQ]) with no missing values. Unknown categories represent both true missingness and NHANES‐coded unknown categories.

### 2.9. Statistical Analysis

The data analysis was performed using Stata 19.0. The descriptive statistics, logistic regression, and Cox regression analyses were performed using the survey weight reported in the NHANES to account for the complex survey design of the pooled data. Specifically, analyses incorporated a complex survey design by applying mobile examination center sampling weights, adjusted for the number of combined survey cycles. Stratification and clustering were accounted for using the appropriate strata and primary sampling unit (PSU) variables. Single‐PSU strata were handled using the certainty option. For subgroup analyses, the subpopulation approach was used to preserve the survey design and weighting structure [30].

We obtained the distribution of study participants according to age, marital status, ethnicity, income, education, smoking status, alcohol usage, physical activity, and obesity for overall complete cases as well as for the subgroups of individuals with CircS, MetS, and AL. We also provided a concise comparison of baseline characteristics between the full MetS/CircS analytic cohort and the AL analytic cohort. Since the three markers depend on age and sex, and mortality incidence also varies by age and sex, a priori, prespecified subgroup analyses by age and sex were performed. Accordingly, the distribution of study characteristics was also evaluated for age and sex subgroups. Based on prior literature and data‐driven analyses [18, 31], we used 70 years as the threshold for age stratification. The incidence of mortality was computed using the unweighted Kaplan–Meier method for descriptive purposes only. To determine the effect of each exposure on survival, we performed a stratified Cox regression analysis with and without adjusting for baseline covariates on the entire cohort. We also tested whether prespecified subgroup analyses of age and sex reflect statistical interactions by including their product terms with each exposure in the statistical analysis. Considering the significance of the interaction between age and each exposure on mortality and sex differences in individual markers, we evaluated the impact of exposures on mortality according to age (< 70 and ≥ 70), sex, and by age and sex. We used the Schoenfeld test and scaled Schoenfeld residuals to systematically assess PH assumptions for all Cox models, including AL, without accounting for survey weights. Since the proportionality hazard assumption in the Cox models was significant for the association of MetS and CircS with survival probability, we performed the stratified Cox regression after dividing the time to incident mortality into three categories (< 50 months, 50 to 100 months, and greater than 100 months). The time interval thresholds for stratified Cox models were chosen based on the pattern of change in the Schoenfeld residuals. The stratified Cox models provide the average hazard ratios (HRs) across Cox models developed on a stratified variable [32]. Time interval‐specific HRs were also computed via standard Cox models to determine the pattern of associations over follow‐up. In a sensitivity analysis, the results were also confirmed using standard Cox models, ignoring the proportional hazards assumption. Furthermore, we also tested the adjusted association between each exposure and mortality after additionally adjusting for obesity to further confirm whether the exposure and outcome association persists beyond obesity. However, the results of these analyses should be interpreted with caution, as an obesity‐related measure, such as waist circumference, is included in the exposure definition and may lead to over‐adjustment concerns.

Since MetS, CircS, and AL cannot be included within the same analytical framework, as these constructs include some common markers and yield collinearity in the analyses, the adjusted effect of each exposure was compared across models with an interaction term between exposure and cohort using survey‐weighted Cox models. Owing to missing data on biomarkers for computing CircS, MetS, and AL, we further validated our primary findings in a sensitivity analysis by restricting the analysis to complete data for CircS, MetS, and AL. To provide a direct effect of each construct on mortality, we created a composite exposure variable with four categories: no exposure, MetS only, primary AL, and primary CircS. Primary AL was defined as high AL in the absence of CircS, regardless of MetS status, whereas primary CircS was defined as the presence of CircS irrespective of MetS or AL. In addition, we evaluated the simultaneous effects of all exposures, ignoring construct overlap. Although these analyses on a restricted dataset allowed us to directly compare the effects of each exposure on mortality within a single model, the results may be biased by collinearity and should be interpreted with caution. The performance of Cox regression models was evaluated using Harrell’s C‐statistic. To further validate the performance of each exposure across models, we reported survey‐weighted prognostic score adjusted effect size and model accuracy as measured with the C‐statistic [33]. The purpose of prognostic score–adjusted comparisons was to evaluate the strength of association rather than the clinical prediction across models. In additional sensitivity analyses, we performed survey‐weighted logistic regression analyses to confirm the findings from the time‐to‐event analysis. HRs (uHR for unadjusted, aHR for adjusted) along with 95% confidence intervals (CIs) and the p‐values were reported for the Cox regression models. The odds ratios (OR) along with 95% CI and the p‐values were reported for the logistic regression models. HRs and ORs for AL are presented comparing for individuals with high AL relative to those with low AL. Statistical analyses were conducted according to statistical writing frameworks and guidance checklists [34, 35].

### 2.10. Ethical Considerations

The NHANES study is approved by the Institutional Review Board (IRB) of the National Center for Health Statistics (NCHS), and consent was obtained from all participants. This study was considered exempt research under 45 CFR § 46.104(d) (4) as it involved only the secondary use of data from a publicly available database in compliance with the Health Insurance Portability and Accountability Act (HIPAA), specifically, 45 CFR § 164.514.

## 3. Results

### 3.1. Study Characteristics

Of the total eligible cohort of 41432 subjects, we excluded 3998 (9.6%) due to missing data on biomarkers of MetS or CircS, whereas 3088 records (10.4%) of 29568 were excluded due to missing data on biomarkers related to AL analysis (Figure S1). Missing covariates were considered as unknown categories and were not excluded from the analyses. Table 1 provides baseline characteristics of the participants according to CircS, MetS, and AL status. Among 37,343 U.S. adults, 11366 (28.22%) subjects had CircS, and 15743 (40.1%) subjects were identified with MetS. The majority of participants were younger (74.5%), non‐Hispanic white (67.1%), and belonged to low‐income (41.8%) groups. Most participants had college or higher education (58.6%) and reported alcohol usage (81.8%). Gender and physical activity were equally distributed. MetS and CircS were commonly observed in individuals of older ages and female gender, smokers, and the obese group. Among 26,480 U.S. adults included in the AL analysis, 4749 (18.4%) individuals had high AL. The high AL was found to be more prevalent in individuals of younger ages, male gender, married status, Hispanic ethnicity, smokers, and alcohol users. The distributions of baseline characteristics were almost identical between the full CircS/MetS analytic cohort and the AL analytic cohort (Table S1).

**TABLE 1:** Prevalence of MetS, CircS, and AL by baseline characteristics, NHANES (2005–18).

Factors	MetS	CircS	AL
	*N* = 15743 (40.1)	*N* = 11366 (28.2)	*N* = 4749 (18.4)
Age (years)			
18–69	12269 (37.6%)	9168 (27.2%)	4327 (19.6%)
70 and above	3474 (58.6%)	2198 (35.3%)	422 (9.4%)
Marital status			
Married	8309 (43.1%)	5650 (28.6%)	2435 (19.1%)
Others	7199 (38.2%)	5567 (29.1%)	2226 (18.1%)
Unknown	235 (13.7%)	149 (8.5%)	88 (7.5%)
Ethnicity			
Hispanic	4305 (39.9%)	3061 (27.7%)	1397 (20.2%)
Non‐Hispanic white	6851 (41.2%)	4863 (28.3%)	2016 (18.4%)
Non‐Hispanic black	3331 (39.8%)	2584 (31.8%)	959 (17.9%)
Others	1256 (32.2%)	858 (23.2%)	377 (15.3%)
Income ($)			
0 to 44999	8823 (43.2%)	6771 (33.0%)	2587 (19.0%)
45000 to 99999	2766 (41.7%)	1908 (38.9%)	899 (20.4%)
100000 and above	2994 (35.5%)	1878 (21.9%)	913 (16.7%)
Unknown	1160 (38.8%)	809 (26.5%)	350 (16.6%)
Education			
Less than 9th grade	2018 (48.4%)	1471 (35.1%)	568 (19.3%)
9th to 12th grade	6454 (43.1%)	4860 (32.0%)	2015 (19.9%)
College or above	7252 (37.5%)	5021 (25.3%)	2164 (17.4%)
Unknown	19 (40.1%)	14 (37.9%)	2 (4.5%)
Smoking			
No	8145 (37.7%)	5575 (25.0%)	2397 (16.9%)
Yes	7415 (44.4%)	5670 (33.0%)	2263 (20.6%)
Unknown	183 (12.5%)	121 (8.1%)	89 (8.3%)
Alcohol			
No	2295 (44.3%)	1592 (29.9%)	533 (15.8%)
Yes	12399 (40.9%)	9189 (29.3%)	3845 (19.2%)
Unknown	1049 (27.6%)	585 (15.0%)	371 (13.6%)
Physical activity			
No	9279 (41.6%)	6620 (29.0%)	2541 (18.5%)
Yes	6462 (38.6%)	4745 (27.4%)	2208 (18.3%)
Unknown	2 (53.2%)	1 (15.8%)	0 (0.0%)
Obesity			
No	5857 (23.1%)	4120 (15.9%)	1775 (10.9%)
Yes	9753 (69.7%)	7149 (49.6%)	2934 (31.3%)
Unknown	133 (30.6%)	97 (21.4%)	40 (15.8%)

*Note:* MetS/CircS analytic cohort included 37,304 participants, and AL analytic cohort included 26,480 participants. Unknown categories represent both true missingness and NHANES‐coded unknown categories. MetS: metabolic syndrome.

Abbreviations: AL, allostatic load; CircS, circadian syndrome; NHANES, National Health and Nutrition Examination Surveys.

The mortality data were recorded for a follow‐up period of 15 years with a median follow‐up time of 7.4 years. A total of 3823 (7.6%) mortality were observed in the eligible study cohort. Higher incidence of mortality was observed in individuals with CircS (4.0%), MetS (3%), and high AL (3%) at 5 years, particularly among the younger participants (age < 70). However, mortality was higher in individuals with no CircS (21.3%), no MetS (25.4%), and low AL (22.7%) among elders (age ≥ 70). In addition, a slight difference in mortality pattern with respect to exposures was observed by sex (Table S2). The weighted percentages of mortality in individuals with CircS (6.7%) were higher in individuals aged < 70 years compared to MetS (5.8%) and AL (5.9%) (Table S3).

### 3.2. Association Between Metabolic Stressors and Mortality in the Entire Cohort

**FIGURE 1:**
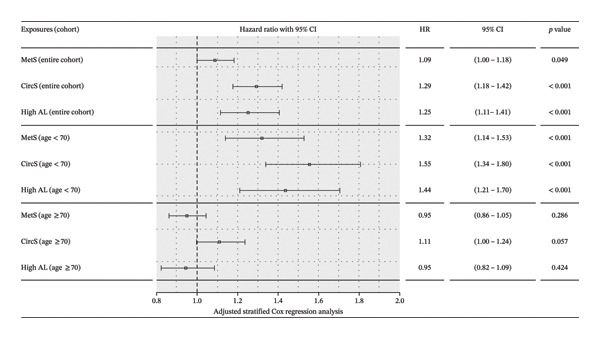
Adjusted effects of CircS, MetS, and high AL on all‐cause mortality in the entire cohort and by age subgroups. Adjusted analyses using survey‐weighted stratified Cox regression were conducted for each exposure separately. Models were adjusted for age, gender, marital status, ethnicity, income, education, smoking, alcohol use, and physical activity. MetS: metabolic syndrome, CircS: Circadian syndrome, AL: allostatic load, HR: hazard ratio, CI: confidence interval, and NHANES: National Health and Nutrition Examination Surveys; MetS/CircS analytic cohort included 37,304 participants, and AL analytic cohort included 26,480 participants.

There was a slight violation of the PH assumption for MetS (*p* = 0.002) and CircS (*p* = 0.007) but not for AL (*p* = 0.16) in adjusted and unadjusted analyses, as confirmed using the Schoenfeld test as well as the Schoenfeld residual plots (Figure S2). In adjusted and stratified Cox regression analyses, we observed a significant association between all three exposures and all‐cause mortality in the entire cohort with CircS (aHR: 1.29; 95% CI 1.18–1.42), MetS (aHR: 1.09; 95% CI 1.00–1.18), and AL (aHR: 1.25; 95% CI 1.11–1.41) (Table 2 and Figure 1). Moreover, the effect of CircS on mortality was significantly higher than that of MetS, but comparable to the effect of AL on mortality (Table 2). In time‐interval‐specific analysis, only CircS was consistently associated with a higher risk of mortality during follow‐up, including time interval < 50 (1.35; 95% CI 1.17–1.55), 50–100 (1.20; 95% CI 1.03–1.40), and > 100 (1.32; 95% CI 1.03–1.67) months (Table S4).

**TABLE 2:** Unadjusted and adjusted effects of MetS, CircS, and AL on all‐cause mortality in the entire cohort and by age groups using the NHANES (2005–18).

Exposure	MetS		CircS		AL	
	HR (95% CI)	*p* value	HR (95% CI)	*p* value	HR (95% CI)	*p* value
Entire cohort						
Unadjusted	1.84 (1.67–2.03)	< 0.001	1.82 (1.62–2.05)	< 0.001	0.98 (0.88–1.10)	0.784
Adjusted	1.09 (1.00–1.18)	0.049	1.29 (1.18–1.42)	< 0.001	1.25 (1.11–1.41)	< 0.001
Comparison	< 0.001^∗^				0.907^∗^	
Age < 70						
Unadjusted	2.06 (1.77–2.40)	< 0.001	2.40 (2.03–2.83)	< 0.001	1.52 (1.27–1.83)	< 0.001
Adjusted	1.32 (1.14–1.53)	< 0.001	1.55 (1.34–1.80)	< 0.001	1.44 (1.21–1.70)	< 0.001
Comparison	0.008^∗^				0.655^∗^	
Age ≥ 70						
Unadjusted	0.88 (0.79–0.98)	0.016	1.06 (0.95–1.18)	0.314	0.87 (0.74–1.02)	0.090
Adjusted	0.95 (0.86–1.05)	0.286	1.11 (1.00–1.24)	0.057	0.95 (0.82–1.09)	0.424
Comparison	0.008^∗^				0.212^∗^	

*Note:* Adjusted analyses using survey‐weighted stratified Cox regression were conducted for each exposure separately. Models were adjusted for age, gender, marital status, ethnicity, income, education, smoking, alcohol use, and physical activity. MetS/CircS analytic cohort included 37,304 participants, and AL analytic cohort included 26,480 participants. MetS: metabolic syndrome.

Abbreviations: AL, allostatic load; CI, confidence interval; CircS, circadian syndrome; HR, hazard ratio; NHANES, National Health and Nutrition Examination Surveys.

^∗^Comparison of adjusted coefficients of MetS and high AL with CircS.

### 3.3. Association Between Metabolic Stressors and Mortality by Age

In the entire cohort, there was an indication of a strong interaction between age and each of the exposures, particularly with age ≥ 70 years (Table S5). The association of CircS (aHR: 1.55; 95% CI 1.34–1.80), MetS (aHR: 1.35; 95% CI 1.14–1.53), and AL (aHR: 1.44; 95% CI 1.21–1.70) with all‐cause mortality was more pronounced among individuals aged < 70 years (Figure 1). However, AL was not significantly associated with all‐cause mortality in the unadjusted analysis (*p* = 0.78). Among individuals aged ≥ 70 years, none of the three metabolic stressors were found to be significantly associated with all‐cause mortality when adjusted for other covariates. CircS was only marginally associated with increased risk of mortality (aHR: 1.11; 95% CI 1.00–1.24, *p* = 0.06). CircS was associated with mortality to a greater extent than MetS in individuals across all ages (Table 2). In time‐interval‐specific analysis, only CircS was consistently associated with a higher risk of mortality during follow‐up among individuals aged < 70 years (Table S4).

### 3.4. Association Between Metabolic Stressors and Mortality by Sex

In the subgroup analyses by sex, all three exposures, including CircS (aHR: 1.37; 95% CI 1.20–1.56, *p* < 0.001), MetS (aHR: 1.12; 95% CI 1.00–1.24, *p* = 0.048), and AL (aHR: 1.28; 95% CI 1.04–1.58, *p* = 0.019), were found to be significantly associated with mortality among males in the entire cohort (Table 3). In the female subgroup analysis, CircS was found to be associated with all‐cause mortality (aHR: 1.21; 95% CI 1.05–1.39), while AL showed borderline association with all‐cause mortality (aHR: 1.19; 95% CI 1.00–1.41, *p* = 0.056). The effect size of CircS in relation to mortality was significantly larger compared to the effect size of MetS, without any differences in the effect sizes of AL in both sexes (Table 3).

**TABLE 3:** Unadjusted and adjusted effects of MetS, CircS, and AL on all‐cause mortality, in the entire cohort and by sex, using the NHANES (2005–18).

Exposure	MetS		CircS		AL	
	HR (95% CI)	*p* value	HR (95% CI)	*p* value	HR (95% CI)	*p* value
Male						
Unadjusted	1.73 (1.54–1.95)	< 0.001	1.81 (1.59–2.06)	< 0.001	0.95 (0.80–1.13)	0.557
Adjusted	1.12 (1.00–1.24)	0.048	1.37 (1.20–1.56)	< 0.001	1.28 (1.04–1.58)	0.019
Comparison	< 0.001^∗^				0.771^∗^	
Female						
Unadjusted	2.00 (1.73–2.30)	< 0.001	1.87 (1.59–2.20)	< 0.001	1.02 (0.87–1.2)	0.79
Adjusted	1.05 (0.93–1.19)	0.413	1.21 (1.05–1.39)	0.010	1.19 (1.00–1.41)	0.056
Comparison	0.060^∗^				0.846^∗^	

*Note:* Adjusted analyses using survey‐weighted stratified Cox regression were conducted for each exposure separately. Models were adjusted for age, gender, marital status, ethnicity, income, education, smoking, alcohol use, and physical activity. MetS/CircS analytic cohort included 37,304 participants, and AL analytic cohort included 26,480 participants. MetS: metabolic syndrome.

Abbreviations: AL, allostatic load; CI, confidence interval; CircS, circadian syndrome; HR, hazard ratio; NHANES, National Health and Nutrition Examination Surveys.

^∗^Comparison of adjusted coefficients of MetS and high AL with CircS.

### 3.5. Association Between Metabolic Stressors and Mortality by Age and Sex

The three‐way interactions between each exposure, age, and sex in relation to mortality were found to be statistically significant, except for AL (Table S5). The association of CircS (aHR: 1.54; 95% CI 1.27–1.85), MetS (aHR: 1.31; 95% CI 1.07–1.60), and AL (aHR: 1.54; 95% CI 1.18–2.00) with the increased risk of mortality remained significant in males with age < 70 years (Table 4). CircS (aHR: 1.21; 95% CI 1.04–1.42) was also associated with mortality in males aged ≥ 70 years. In the female subgroup analysis, the CircS (aHR: 1.55; 95% CI 1.21–1.98) was found to be associated with all‐cause mortality in the subgroup with age < 70 years. The MetS was also found to be significant with all‐cause mortality only in the subgroup of females aged < 70 years (aHR: 1.32; 95% CI 1.04–1.67). However, a marginally significant association between the AL and all‐cause mortality (HR 1.28; 95% CI 0.99–1.66, *p* = 0.061) was found among females aged < 70 years in the adjusted stratified Cox regression analysis (Table 4).

**TABLE 4:** Unadjusted and adjusted effects of MetS, CircS, and AL with all‐cause mortality by age and sex, NHANES (2005–18).

	Male		Female	
	HR (95% CI)	*p* value	HR (95% CI)	*p* value
Association of MetS with mortality				
Age < 70				
Unadjusted	1.90 (1.55–2.33)	< 0.001	2.42 (1.93–3.04)	< 0.001
Adjusted	1.31 (1.07–1.60)	0.010	1.32 (1.04–1.67)	0.023
Age ≥ 70				
Unadjusted	0.92 (0.82–1.04)	0.171	0.87 (0.74–1.02)	0.084
Adjusted	0.98 (0.87–1.09)	0.655	0.94 (0.81–1.09)	0.384
Association of CircS with mortality				
Age < 70				
Unadjusted	2.27 (1.88–2.74)	< 0.001	2.76 (2.14–3.55)	< 0.001
Adjusted	1.54 (1.27–1.85)	< 0.001	1.55 (1.21–1.98)	0.001
Age ≥ 70				
Unadjusted	1.23 (1.05–1.44)	0.010	0.98 (0.84–1.15)	0.804
Adjusted	1.21 (1.04–1.42)	0.016	1.05 (0.90–1.23)	0.527
Association of AL with mortality				
Age < 70				
Unadjusted	1.46 (1.15–1.86)	0.002	1.53 (1.17–1.99)	0.002
Adjusted	1.54 (1.18–2.00)	0.002	1.28 (0.99–1.66)	0.061
Age ≥ 70				
Unadjusted	0.82 (0.65–1.04)	0.107	0.94 (0.74–1.18)	0.583
Adjusted	0.85 (0.69–1.06)	0.146	1.00 (0.81–1.24)	0.997

*Note:* Adjusted analyses using survey‐weighted stratified Cox regression were conducted for each exposure separately. Models were adjusted for age, gender, marital status, ethnicity, income, education, smoking, alcohol use, and physical activity. MetS/CircS analytic cohort included 37,304 participants, and AL analytic cohort included 26,480 participants. MetS: metabolic syndrome.

Abbreviations: AL, allostatic load; CI, confidence interval; CircS, circadian syndrome; HR, hazard ratio; NHANES, National Health and Nutrition Examination Surveys.

### 3.6. Sensitivity Analysis for the Association Between Metabolic Stressors and Mortality in the Entire Cohort and by Age and Sex

Compared to MetS and AL, the association between CircS and all‐cause mortality was found to be stronger in the entire cohort as well as in the subgroup of individuals aged < 70, irrespective of sex. These associations remained unchanged in standard Cox model analyses, despite violating the proportionality assumption (Table S6). After further adjustment for obesity, CircS was significantly associated with a higher risk of mortality in both age groups, to a greater extent than MetS or AL, in stratified Cox analyses (Table S7) and age‐ and sex‐stratified analyses (Table S8). In all sensitivity analyses without adjustment for obesity, these findings were unchanged and aligned with stratified or unstratified Cox analyses, even after analyzing data with logistic regression models (Tables S9‐S10). Furthermore, these associations remained unchanged in sensitivity analyses restricted to participants with complete data on markers for computing CircS, MetS, and AL (Tables S11 and S12). In composite exposure analysis, CircS was only associated with increased risk of all‐cause mortality (Table S13). Simultaneous inclusion of all exposures in one model showed that CircS was only associated with mortality in both age groups (Table S14). The model accuracy, as estimated with Harrell’s C‐statistic, suggested no significant difference in predictive ability of the models with CircS (86.1%), MetS (86.1%), and AL (86.6%) when adjusted for other covariates for the entire cohort. Similar results were observed for the models fitted on subgroups (Table S15). There were no incremental changes in prediction accuracy when any of these constructs were added to a base model including only covariates, particularly for age < 70 years (Table S16). These findings indicate that discrimination is broadly similar across constructs in adjusted models in the entire cohort and age < 70 years. However, prognostic score‐balanced models showed slightly higher predictive accuracy for CircS compared to MetS or AL in stratified Cox regression analyses, particularly in individuals aged < 70 years (Table S17).

## 4. Discussion

This study was conducted to evaluate the association of three competing metabolic health stressors with all‐cause mortality among U.S. adults, using data from seven cycles of the NHANES. Interaction with age notably influenced this association for all three stressors. The findings indicate a significant positive association between all three markers and all‐cause mortality in the entire cohort as well as in adults aged < 70. The CircS showed a higher impact on all‐cause mortality compared to the other two markers after adjusting for all sociodemographic covariates. These associations were consistently observed in all the sensitivity analyses. Although the predictive ability of mortality did not differ according to the three metabolic stressors, reflecting discrimination is broadly similar across constructs in adjusted models, the CircS had a stronger association with all‐cause mortality than MetS for adults, irrespective of age and sex groups.

The findings are consistent with the research conducted to evaluate the individual components of metabolic health stressors, with a positive relationship with all‐cause mortality. Like our study, another study also showed an association of MetS with an increased risk of all‐cause mortality [36]. A meta‐analysis confirmed high AL as a risk factor for all‐cause mortality in addition to CVD mortality [18]. Although a study yielded high AL as a strong predictor of all‐cause and CVD mortality in a dose–response analysis adjusting for demographic and clinical covariates, our study did not find a significant association of AL with all‐cause mortality in older adults (age ≥ 70 years). Furthermore, consistent with our study observation, a study yielded no differences in the predictive performance of CircS and MetS for all‐cause mortality [10, 37]. In addition, a comparison of CircS and MetS for predicting CVD mortality did not provide evidence of a significant difference in the predictive ability of the two metabolic stressors [10]. In parallel, no differences in predictive performance for CVD mortality were noted with high AL as well [38]. These observations, in line with our study findings, suggest that any of these three markers can be used for mortality risk stratification. However, our study identified that CircS was associated with all‐cause mortality to a greater extent than MetS or AL and yielded a slightly higher predictive accuracy in prognostic score–adjusted analysis, particularly for individuals aged < 70 years. This suggests that CircS is more associated with mortality than other markers. This is likely due to additional factors of depression and sleep involved in the measurement of CircS. Depression and sleep quality have been noted to be markers for all‐cause mortality [39].

Our study observed a notable difference in the association between markers of metabolic stressors and all‐cause mortality by age groups. In our study, we observed that all markers were associated with all‐cause mortality among younger individuals aged < 70 years. However, metabolic stressors, particularly, MetS and AL were not found to be consistently associated with mortality in the subgroup of individuals aged ≥ 70 years. Only CircS showed a marginal association with increased risk of mortality in the entire cohort, as well as a stronger association with mortality in male individuals aged > 70 years, indicating CircS may be associated with a higher risk of mortality, particularly in men across all ages. Similar to our findings, another study identified a significant interaction between age and CircS for all‐cause mortality. In addition, the metabolic score for visceral fat with all‐cause mortality was positively associated with CVD mortality and cancer mortality in adults aged < 65 [31]. A meta‐analysis [18] reported a stronger association between AL and mortality in younger individuals (age < 65). These findings confirmed the association between metabolic stressors and all‐cause mortality in younger ages, but no or attenuated association in older ages. There are several possible explanations for this finding in our study, including survivor bias, competing risks, higher comorbidity burden reducing discrimination, and potential misclassification from single‐time‐point exposure measurement. Participants who survived to age 70 may represent a more resilient subset of the cohort due to survivor bias, potentially reflecting favorable lifestyle factors, genetic advantages, or greater access to health care. The potential for additional increases in mortality risk associated with these metabolic stressors may be limited among participants aged ≥ 70 with higher baseline mortality, owing to ceiling/threshold effects. Reverse causation/frailty‐related weight and metabolic changes may further contribute to biased or attenuated associations. This is consistent with our previous studies, in which metabolic‐associated disorders [25, 40], including the obesity paradox [41], were more strongly associated with outcomes in younger individuals than in older individuals. This may be likely due to limited comorbidities in younger ages compared to older ages. There is another possibility that these metabolic markers may lead to CVD and other diseases, which subsequently increases the propensity of mortality through competing risks and shifting causes of death among older individuals. A study using NHANES data analysis revealed that PhenoAgeAccel mediated the association between CircS and all‐cause mortality [42]. These age‐based differences indicate that screening of metabolic stressors may need to be tailored by age, with greater emphasis on early identification in younger populations. In our study, the absence of a modifying effect of sex on the association of metabolic stressors with all‐cause mortality indicated a universal role of all three metabolic health markers in the risk of all‐cause mortality regardless of sex. Although no study has evaluated the sex differences in the associations between CircS and AL and all‐cause mortality, a study on nondiabetic individuals showed a similar association between MetS and all‐cause mortality in both sexes, consistent with our study findings [3]. However, a systematic review and meta‐analysis revealed sex differences in the association between the MetS and cause‐specific mortalities [43].

The underlying mechanism between metabolic markers and mortality is multifactorial. Untreated metabolic abnormalities lead to multiple chronic diseases and inflammatory disorders that put individuals at risk of mortality [44]. Poor metabolic health has been known to affect molecular and cellular changes and their characteristics that show long‐term adverse effects on morbidity and mortality, even if the metabolic health returns to its normal state [45]. Accumulating evidence indicates that metabolic stressors activate multiple systems, including the hypothalamic–pituitary–adrenocortical (HPA) axis, central nervous system (CNS), and sympatho‐adrenomedullary (SAM) system, simulating reactive oxygen species, oxidative stress, and epigenetic regulation that subsequently modify the physiologic, behavioral, and metabolic response [35, 46, 47]. Further, disturbance in the circadian rhythm, influenced by lifestyle factors, such as sleep loss and working pattern, leads to misaligned physiological processes, affecting lipid metabolism and glucose, and causes the risk of obesity, type 2 diabetes, and CVD [7].

## 5. Strengths and Limitations

One of the primary limitations of this study is the unavailability of data on AL for all cycles, making an inappropriate comparison of the effect size with the other two metabolic health stressors. This also limits the generalizability of AL findings to the U.S. population. However, the sociodemographic and clinical characteristics of the AL study sample are similar to data available for MetS and CircS. Our restricted data analysis, which included all exposure data, confirmed the robustness of the findings reported in the primary data analysis. Nonetheless, the main finding of the AL is consistent with other studies conducted on CVD and all‐cause mortality. In addition, there is a possibility that we might have missed some additional confounders, including medications, in our study. Owing to one‐time data collection of all covariates, we could not account for the changes in the metabolic stressors and their impacts on all‐cause mortality. The reliance on a single measurement of metabolic stressors may limit our understanding of its variability and association with mortality in younger individuals. Additionally, dependence on self‐reported data on some components, such as sleep duration and physical activity, may cause recall bias and yield biased associations. Some results from the sensitivity analyses, such as obesity‐adjustment models, combined‐construct models, and the simultaneous inclusion of all exposures in adjustment models, should be interpreted with caution owing to over‐adjustment and collinearity. Nonetheless, all these models yielded highly consistent findings, as reported in our primary analyses. Furthermore, some associations were found to be only marginally significant, particularly for MetS and AL in stratified analyses by age and sex.

Many studies showed a positive association of individual markers of metabolic stressors on different morbidity and mortality events, such as the impact of the hemoglobin glycation index on CVD and all‐cause mortality [47, 48]. Instead of relying on a single symptom of metabolic stressors, a combined biomarker of metabolic stressors, such as CircS, MetS, and AL, may be more useful for risk stratification and disease management. Our study evaluated and compared the impact of these three metabolic stressors using a large, pooled dataset from 7 cycles of NHANES. To our knowledge, this study is the first to report the relative impact of metabolic stressors measured for screening purposes on the risk of all‐cause mortality in relatively healthy individuals using a U.S. representative cohort with a median follow‐up time of 7.5 years. The study also covered the possible interactions of these metabolic stressors with age and sex and described the associations by subgroup analyses. Our comprehensive data analysis, including multiple sensitivity analyses, yielded consistent results, attesting to the robustness of the findings reported in our study. The implications of these findings are significant, as they underscore the importance of monitoring various risk factors and developing interventions to reduce their impact on human health.

## 6. Conclusions

All three metabolic stressors, including MetS, CircS, and high AL, were found to be associated with a higher risk of mortality, particularly among individuals aged < 70 years. The strength of association between CircS and all‐cause mortality was stronger compared to the other two metabolic stressors, irrespective of sex. Our findings indicate that metabolic biomarkers, specifically CircS, which includes depression and sleep quality, along with MetS markers, may help refine risk stratification and prognostic evaluation among adults aged less than 70 years.

## Author Contributions

Conceptualization: Shakeel Ahmed and Alok Kumar Dwivedi; methodology: Shakeel Ahmed, Pallavi Dubey, and Alok Kumar Dwivedi; software: Shakeel Ahmed and Alok Kumar Dwivedi; investigation: Shakeel Ahmed, Alok Kumar Dwivedi, and Pallavi Dubey; writing–original draft preparation: Shakeel Ahmed; writing–review and editing: Randi E. Foraker and Alok Kumar Dwivedi; and supervision: Randi E. Foraker and Alok Kumar Dwivedi.

## Funding

The authors declare that they did not receive funding.

## Disclosure

All authors have read and agreed to the published version of the manuscript.

## Conflicts of Interest

The authors declare no conflicts of interest.

## Supporting Information

Additional supporting information can be found online in the Supporting Information section.

## Supporting information


**Supporting Information** Description of supporting information: Figure S1: Step‐by‐step selection of eligible participants for the analysis of (A) MetS and CircS, and (B) AL. Figure S2: Test of the proportionality hazards assumption for (A) MetS, (B) CircS, and (C) AL in unweighted unadjusted Cox analysis; (D) MetS, (E) CircS, and (F) AL in unweighted adjusted Cox analysis. Table S1: Comparison of baseline characteristics in the full MetS/CircS analytic cohort and the AL analytic cohort. Table S2: Unweighted descriptive incidence of mortality at 5 years by MetS, CircS, and AL in the entire cohort, by age and sex, using the NHANES (2005–18). Table S3: Survey‐weighted percentage of mortality by MetS, CircS, and AL in the entire cohort, by age and sex in NHANES (2005–18). Table S4: Time interval‐specific adjusted effects of MetS, CircS, and AL with all‐cause mortality in the entire cohort and by age groups in NHANES (2005–18). Table S5: Interactions of continuous and binary age, sex, with CircS, MetS, and AL for all‐cause mortality using Cox and logistic regression analyses, NHANES (2005–18). Table S6: Adjusted associations of MetS, CircS, and AL with all‐cause mortality using standard Cox models, NHANES (2005–18). Table S7: Adjusted effects of MetS, CircS, and AL on all‐cause mortality using stratified Cox regression analyses, after additionally adjusting for obesity, NHANES (2005–18). Table S8: Adjusted associations of MetS, CircS, and AL with all‐cause mortality by age and sex, additionally adjusted with obesity using stratified Cox regression analyses, NHANES (2005–18). Table S9: Unadjusted and adjusted associations of MetS, CircS, and AL with all‐cause mortality using logistic regression analyses, NHANES (2005–18). Table S10: Unadjusted and adjusted associations of MetS, CircS, and AL with all‐cause mortality by age and sex using logistic regression analyses, NHANES (2005–10 and 2015–18). Table S11: Unadjusted and adjusted effects of MetS, CircS, and AL on all‐cause mortality in sensitivity analyses restricted to complete data on all markers for computing MetS, CircS, and AL in the entire cohort and by age groups using the NHANES (2005–10 and 2015–18). Table S12: Unadjusted and adjusted effects of MetS, CircS, and AL on all‐cause mortality by sex in sensitivity analyses restricted to complete data on all markers for computing MetS, CircS, and AL using the NHANES (2005–10 and 2015–18). Table S13: Adjusted effect of composite exposure variable on all‐cause mortality in sensitivity analyses restricted to complete data on all markers for computing MetS, CircS, and AL in the entire cohort and by age groups using the NHANES (2005–10 and 2015–18). Table S14: Simultaneous adjusted effects of all exposure variables on all‐cause mortality after ignoring overlapping across exposures in sensitivity analyses restricted to complete data on all markers for computing MetS, CircS, and AL in the entire cohort and by age groups using the NHANES (2005–10 and 2015–18). Table S15: Performance metric (Harrell’s c‐statistic) for Cox models with each exposure. Table S16: Incremental differences in prediction accuracies by inclusion of each exposure in adjusted analysis compared to a base model including only baseline covariates in the entire cohort and by age groups using the NHANES (2005–18). Table S17: Prognostic score adjusted effects of MetS, CircS, and AL on all‐cause mortality in the entire cohort and individuals aged < 70 years, using stratified Cox regression analyses, NHANES (2005–18).

## Data Availability

The data used in this study are publicly available from the NHANES conducted by the Centers for Disease Control and Prevention (CDC). All data files can be accessed at https://www.cdc.gov/nchs/nhanes/index.htm. No special access or permissions are required.
