# Supplementary material for: Comparative Evaluation of Metabolic Syndrome, Circadian Syndrome, and Allostatic Load Measures on All‐Cause Mortality in U.S. Males and Females
Source: Int J Endocrinol. 2026 May 11;2026:7064400. doi: 10.1155/ije/7064400 (PMC13161462; doi:10.1155/ije/7064400)
Supplement: Supplementary file 1 — Supporting Information Description of supporting information: Figure S1: Step‐by‐step selection of eligible participants for the analysis of (A) MetS and CircS, and (B) AL. Figure S2: Test of the proportionality hazards assumption for (A) MetS, (B) CircS, and (C) AL in unweighted unadjusted Cox analysis; (D) MetS, (E) CircS, and (F) AL in unweighted adjusted Cox analysis. Table S1: Comparison of baseline characteristics in the full MetS/CircS analytic cohort and the AL analytic cohort. Table S2: Unweighted descriptive incidence of mortality at 5 years by MetS, CircS, and AL in the entire cohort, by age and sex, using the NHANES (2005–18). Table S3: Survey‐weighted percentage of mortality by MetS, CircS, and AL in the entire cohort, by age and sex in NHANES (2005–18). Table S4: Time interval‐specific adjusted effects of MetS, CircS, and AL with all‐cause mortality in the entire cohort and by age groups in NHANES (2005–18). Table S5: Interactions of continuous and binary age, sex, with CircS, MetS, and AL for all‐cause mortality using Cox and logistic regression analyses, NHANES (2005–18). Table S6: Adjusted associations of MetS, CircS, and AL with all‐cause mortality using standard Cox models, NHANES (2005–18). Table S7: Adjusted effects of MetS, CircS, and AL on all‐cause mortality using stratified Cox regression analyses, after additionally adjusting for obesity, NHANES (2005–18). Table S8: Adjusted associations of MetS, CircS, and AL with all‐cause mortality by age and sex, additionally adjusted with obesity using stratified Cox regression analyses, NHANES (2005–18). Table S9: Unadjusted and adjusted associations of MetS, CircS, and AL with all‐cause mortality using logistic regression analyses, NHANES (2005–18). Table S10: Unadjusted and adjusted associations of MetS, CircS, and AL with all‐cause mortality by age and sex using logistic regression analyses, NHANES (2005–10 and 2015–18). Table S11: Unadjusted and adjusted effects of MetS, CircS, and AL on all‐cause [file IJE-2026-7064400-s001.docx]

**Figure S1. Step-by-step selection of eligible participants for the analysis of (A) MetS and CircS, and (B) AL. (MetS: Metabolic syndrome, CircS: Circadian syndrome, and AL: Allostatic load; MetS/CircS analytic cohort included 37304 participants, and AL analytic cohort included 26480 participants)**


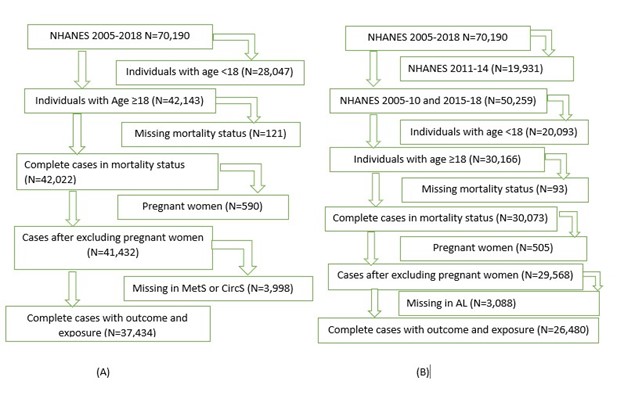


**Figure S2. Test of the proportionality hazards assumption for (A) MetS, (B) CircS, and (C) AL in unweighted unadjusted Cox analysis; (D) MetS, (E) CircS, and (F) AL in unweighted adjusted Cox analysis. (MetS: Metabolic syndrome, CircS: Circadian syndrome, and AL: Allostatic load; MetS/CircS analytic cohort included 37304 participants, and AL analytic cohort included 26480 participants)**

**
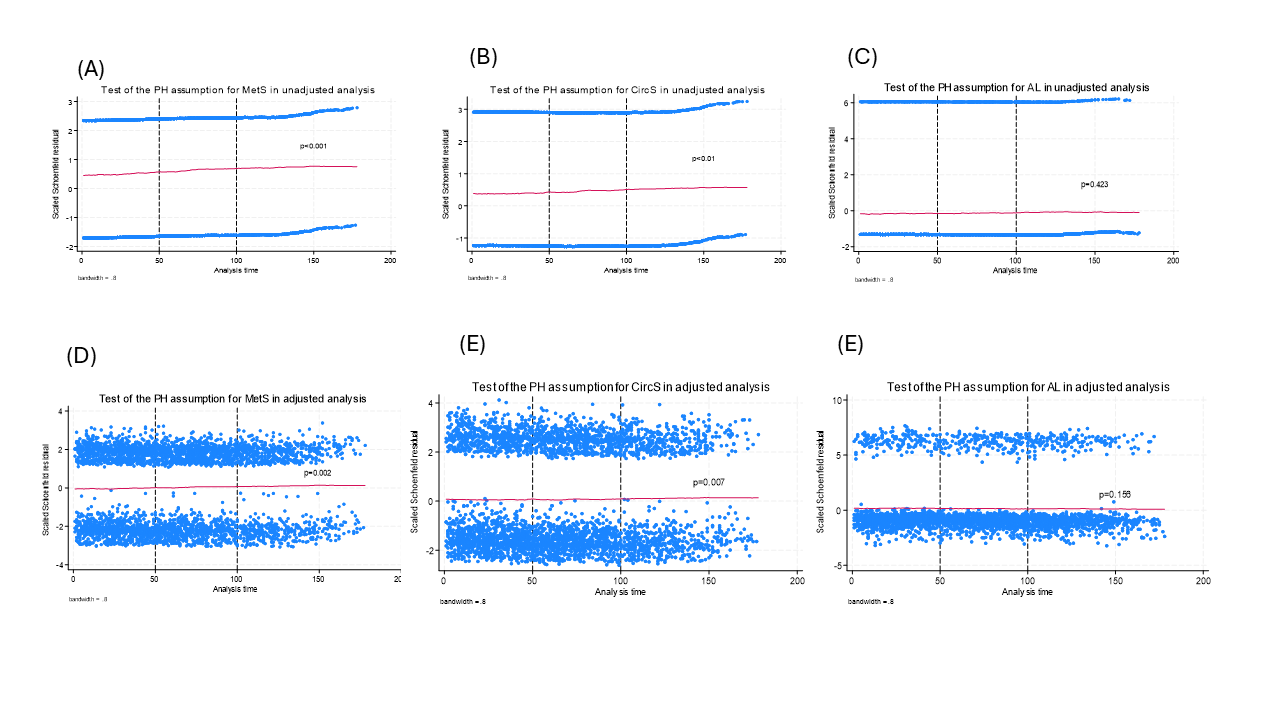
**

**Table S1. Comparison of baseline characteristics in the full MetS/CircS analytic cohort and the AL analytic cohort**

|  | **MetS/CircS (N=37,434)** | | **AL (N=26480)** | |
| --- | --- | --- | --- | --- |
| **Age (years)** |  |  |  |  |
| 18-69 | 25361 | 74.5 | 17755 | 74.6 |
| 70 and above | 12073 | 25.5 | 8725 | 25.4 |
| **Gender** |  |  |  |  |
| Male | 18433 | 48.7 | 13084 | 48.8 |
| Female | 19001 | 51.3 | 13396 | 51.2 |
| **Marital status** |  |  |  |  |
| Married | 18250 | 53.7 | 13030 | 54.2 |
| Others | 17583 | 43.5 | 12434 | 43.3 |
| Unknown | 1601 | 2.8 | 1016 | 2.5 |
| **Ethnicity** |  |  |  |  |
| Hispanic | 9681 | 14.3 | 7297 | 14.1 |
| Non-Hispanic white | 15560 | 67.1 | 11192 | 67.4 |
| Non-Hispanic black | 7999 | 10.9 | 5471 | 10.9 |
| Others | 4194 | 7.6 | 2520 | 7.6 |
| **Income** |  |  |  |  |
| 0 to 44999 | 19696 | 41.8 | 13858 | 40.9 |
| 45000 to 99999 | 6520 | 19.8 | 4708 | 20.1 |
| 100000 and above | 8360 | 32 | 5804 | 32.4 |
| Unknown | 2858 | 6.3 | 2110 | 6.6 |
| **Education** |  |  |  |  |
| less than 9th grade | 3905 | 5.5 | 2995 | 5.7 |
| 9th to 12th grade | 15133 | 35.9 | 10867 | 36.5 |
| college or above | 18356 | 58.6 | 12587 | 57.7 |
| Unknown | 40 | 0.1 | 31 | 0.1 |
| **Smoking** |  |  |  |  |
| No | 20197 | 54.4 | 14074 | 53.9 |
| Yes | 15934 | 43.8 | 11386 | 44.2 |
| Unknown | 1303 | 1.8 | 1020 | 1.9 |
| **Alcohol** |  |  |  |  |
| No | 4935 | 10.2 | 3288 | 9.7 |
| Yes | 28639 | 81.8 | 20221 | 81.8 |
| Unknown | 3860 | 8 | 2971 | 8.5 |
| **Physical activity** |  |  |  |  |
| No | 21079 | 51.4 | 14193 | 48 |
| Yes | 16351 | 48.6 | 12285 | 52 |
| Unknown | 4 | 0 | 2 | 0 |
| **Obesity** |  |  |  |  |
| No | 23213 | 62.7 | 16337 | 62.6 |
| Yes | 13784 | 36.4 | 9880 | 36.7 |
| Unknown | 437 | 0.9 | 263 | 0.7 |

Data are expressed with counts and percentages. MetS: Metabolic syndrome, CircS: Circadian syndrome, and AL: Allostatic load, NHANES: National Health and Nutrition Examination Surveys. MetS/CircS analytic cohort included 37304 participants, and AL analytic cohort included 26480 participants. Unknown categories represent both true missingness and NHANES-coded unknown categories.

**Table S2. Unweighted descriptive incidence of mortality at 5 years by MetS, CircS, and AL in the entire cohort, by age and sex, using the NHANES (2005-18)**

|  | **Entire** | | | | **Male** | | | | **Female** | | |
| --- | --- | --- | --- | --- | --- | --- | --- | --- | --- | --- | --- |
|  | **IR** | **95%CI** | | **IR** | | **95%CI** | | **IR** | | **95%CI** | |
| **Entire cohort** | | | | | | | | | | | |
| **MetS** |  |  |  |  | |  |  |  | |  |  |
| No | 0.05 | 0.04 | 0.05 | 0.06 | | 0.05 | 0.06 | 0.03 | | 0.03 | 0.04 |
| Yes | 0.07 | 0.07 | 0.08 | 0.09 | | 0.08 | 0.10 | 0.06 | | 0.05 | 0.07 |
| **CircS** |  |  |  |  | |  |  |  | |  |  |
| No | 0.05 | 0.05 | 0.05 | 0.06 | | 0.06 | 0.06 | 0.04 | | 0.04 | 0.04 |
| Yes | 0.07 | 0.07 | 0.08 | 0.09 | | 0.08 | 0.10 | 0.06 | | 0.05 | 0.06 |
| **AL** |  |  |  |  | |  |  |  | |  |  |
| No | 0.06 | 0.06 | 0.06 | 0.07 | | 0.07 | 0.08 | 0.05 | | 0.04 | 0.05 |
| Yes | 0.05 | 0.04 | 0.05 | 0.06 | | 0.05 | 0.07 | 0.03 | | 0.03 | 0.04 |
| **Age<70** | | | | | | | | | | | |
| **MetS** |  |  |  |  | |  |  |  | |  |  |
| No | 0.02 | 0.02 | 0.02 | 0.02 | | 0.02 | 0.03 | 0.01 | | 0.01 | 0.01 |
| Yes | 0.03 | 0.03 | 0.04 | 0.04 | | 0.04 | 0.05 | 0.03 | | 0.02 | 0.03 |
| **CircS** |  |  |  |  | |  |  |  | |  |  |
| No | 0.02 | 0.02 | 0.02 | 0.02 | | 0.02 | 0.03 | 0.01 | | 0.01 | 0.01 |
| Yes | 0.04 | 0.03 | 0.04 | 0.05 | | 0.04 | 0.06 | 0.03 | | 0.03 | 0.04 |
| **AL** |  |  |  |  | |  |  |  | |  |  |
| No | 0.02 | 0.02 | 0.02 | 0.03 | | 0.02 | 0.03 | 0.02 | | 0.01 | 0.02 |
| Yes | 0.03 | 0.03 | 0.04 | 0.04 | | 0.04 | 0.05 | 0.02 | | 0.01 | 0.03 |
| **Age≥70** | | | | | | | | | | | |
| **MetS** |  |  |  |  | |  |  |  | |  |  |
| No | 0.25 | 0.24 | 0.27 | 0.27 | | 0.25 | 0.30 | 0.23 | | 0.20 | 0.26 |
| Yes | 0.21 | 0.20 | 0.23 | 0.26 | | 0.24 | 0.28 | 0.18 | | 0.16 | 0.19 |
| **CircS** |  |  |  |  | |  |  |  | |  |  |
| No | 0.24 | 0.23 | 0.26 | 0.26 | | 0.24 | 0.28 | 0.21 | | 0.19 | 0.24 |
| Yes | 0.21 | 0.20 | 0.23 | 0.28 | | 0.25 | 0.31 | 0.17 | | 0.15 | 0.19 |
| **AL** |  |  |  |  | |  |  |  | |  |  |
| No | 0.23 | 0.21 | 0.24 | 0.27 | | 0.25 | 0.29 | 0.19 | | 0.17 | 0.21 |
| Yes | 0.19 | 0.16 | 0.24 | 0.23 | | 0.18 | 0.30 | 0.16 | | 0.11 | 0.22 |

IR: Incidence rate; MetS: Metabolic syndrome, CircS: Circadian syndrome, and AL: Allostatic load, NHANES: National Health and Nutrition Examination Surveys. MetS/CircS analytic cohort included 37304 participants, and AL analytic cohort included 26480 participants. Unweighted incidence may not reflect U.S.-representative risk under the NHANES sampling design.

**Table S3.** **Survey-weighted percentage of mortality by MetS, CircS, and AL in the entire cohort, by age and sex in NHANES (2005-18)**

|  | **Total** | **Age<70** | **Age≥70** |
| --- | --- | --- | --- |
| **Entire cohort for MetS/CircS** | 3823 (7.6) | 1478 (3.9) | 2445 (34.8) |
| **Gender** |  |  |  |
| Male | 2202 (4.0) | 899 (4.8) | 1303 (37.7) |
| Female | 1621 (3.6) | 579 (3.0) | 1042 (32.7) |
| **MetS** |  |  |  |
| No | 1717 (5.7) | 646 (2.8) | 1071 (37.2) |
| Yes | 2106 (10.6) | 832 (5.8) | 1274 (33.2) |
| **CircS** |  |  |  |
| No | 2266 (6.3) | 769 (2.9) | 1497 (34.2) |
| Yes | 1557 (11.1) | 709(6.7) | 848 (35.9) |
| **Entire cohort for AL** | 2881 (7.9) | 1101 (4.0) | 1780 (35.4) |
| **AL** |  |  |  |
| No | 2415 (7.9) | 797 (3.6) | 1618 (35.8) |
| Yes | 466 (7.5) | 304 (5.9) | 162 (31.9) |

MetS: Metabolic syndrome, CircS: Circadian syndrome, and AL: Allostatic load, NHANES: National Health and Nutrition Examination Surveys. MetS/CircS analytic cohort included 37304 participants, and AL analytic cohort included 26480 participants.

**Table S4.** **Time interval-specific adjusted effects of MetS, CircS, and AL with all-cause mortality in the entire cohort and by age groups in NHANES (2005-18)**

| **Exposure** | **MetS** | | **CircS** | | **AL** | |
| --- | --- | --- | --- | --- | --- | --- |
| **Time(months)** | **HR (95%CI)** | **p value** | **HR (95%CI)** | **p value** | **HR (95%CI)** | **p value** |
| **Entire cohort** | | | | | | |
| **<50** | 0.97(0.83–1.12) | 0.657 | 1.35(1.17–1.55) | <0.001 | 1.36(1.09–1.70) | 0.007 |
| **50-100** | 1.12(0.97–1.28) | 0.111 | 1.20(1.03–1.40) | 0.017 | 1.15(0.96–1.37) | 0.124 |
| **>100** | 1.23(0.98–1.53) | 0.069 | 1.32(1.03–1.67) | 0.026 | 1.17(0.94–1.46) | 0.149 |
| **Age<70** | | | | | | |
| **<50** | 1.19(0.93–1.52) | 0.173 | 1.57(1.24–1.99) | <0.001 | 1.64(1.21–2.22) | 0.002 |
| **50-100** | 1.42(1.12–1.80) | 0.004 | 1.57(1.25–1.98) | <0.001 | 1.22(0.92–1.62) | 0.172 |
| **>100** | 1.36(0.98–1.88) | 0.063 | 1.52(1.12–2.08) | 0.009 | 1.35(1.03–1.76) | 0.028 |
| **Age≥70** | | | | | | |
| **<50** | 0.86(0.73–1.01) | 0.071 | 1.22(1.04–1.43) | 0.013 | 1.04(0.80–1.34) | 0.782 |
| **50-100** | 0.93(0.79–1.10) | 0.386 | 0.98(0.81–1.19) | 0.862 | 1.00(0.78–1.28) | 0.995 |
| **>100** | 1.13(0.91–1.41) | 0.249 | 1.13(0.90–1.44) | 0.288 | 0.85(0.64–1.15) | 0.292 |

Adjusted analyses using survey-weighted Cox regression were conducted for each exposure separately. Models were adjusted for age, gender, marital status, ethnicity, income, education, smoking, alcohol use, and physical activity. MetS: Metabolic syndrome, CircS: Circadian syndrome, and AL: Allostatic load, HR: Hazard ratio; CI: Confidence interval; NHANES: National Health and Nutrition Examination Surveys; MetS/CircS analytic cohort included 37304 participants, and AL analytic cohort included 26480 participants.

**Table S5.** **Interactions of continuous and binary age, sex, with CircS, MetS, and AL for all-cause mortality using Cox and logistic regression analyses, NHANES (2005-18)**

|  | **Cox Regression** |  | **Logistic Regression** |  |
| --- | --- | --- | --- | --- |
| **Interactions** | **HR (95%CI)** | **p-value** | **OR (95%CI)** | **p-value** |
| **Three-way interaction** | | | | |
| Age, sex, and MetS | 0.98(0.96–0.99) | 0.01 | 0.98(0.96–1.00) | 0.015 |
| Age, sex, and CircS | 0.98(0.97–1.00) | 0.025 | 0.98(0.96–1.00) | 0.017 |
| Age, sex, and AL | 1.00(0.98–1.02) | 0.671 | 0.99(0.97–1.02) | 0.607 |
| **Two-way interaction** | | | | |
| Age and MetS | 0.99(0.99–1.00) | 0.045 | 0.99(0.99–1.00) | 0.263 |
| Sex and MetS | 1.14(0.97–1.36) | 0.12 | 1.17(0.98–1.39) | 0.083 |
| Age and CircS | 0.99(0.98–0.99) | 0.001 | 0.99(0.98–1.00) | 0.038 |
| Sex and CircS | 1.03(0.87–1.23) | 0.717 | 1.06(0.88–1.28) | 0.518 |
| Age and AL | 0.98(0.97–0.99) | <0.001 | 0.98(0.97–0.99) | <0.001 |
| Sex and AL | 1.03(0.79–1.34) | 0.853 | 1.02(0.78–1.33) | 0.902 |
| **Two-way interaction with binary age** | | | | |
| Age≥70 and MetS | 0.42(0.35–0.51) | <0.001 | 0.39(0.31–0.48) | <0.001 |
| Age≥70 and CircS | 0.45(0.37–0.54) | <0.001 | 0.45(0.36–0.55) | <0.001 |
| Age≥70 and AL | 0.56(0.42–0.73) | <0.001 | 0.50(0.36–0.69) | <0.001 |

Survey-weighted Cox regression was conducted for each exposure separately; MetS: Metabolic syndrome, CircS: Circadian syndrome, and AL: Allostatic load, HR: Hazard ratio; OR: Odds ratio; CI: Confidence interval; NHANES: National Health and Nutrition Examination Surveys; MetS/CircS analytic cohort included 37304 participants, and the AL analytic cohort included 26480 participants.

**Table S6.** **Adjusted associations of MetS, CircS, and AL with all-cause mortality using standard Cox models, NHANES (2005-18)**

|  | **MetS** | | **CircS** | | **AL** | |
| --- | --- | --- | --- | --- | --- | --- |
|  | **HR (95%CI)** | **p-value** | **HR (95%CI)** | **p-value** | **HR (95%CI)** | **p-value** |
| **Entire cohort** | 1.07(0.98–1.16) | 0.123 | 1.16(1.07–1.26) | 0.001 | 1.20(1.07–1.35) | 0.003 |
| **Age<70** | 1.33(1.15–1.53) | <0.001 | 1.47(1.28–1.68) | <0.001 | 1.45(1.25–1.69) | <0.001 |
| **Age≥70** | 0.94(0.85–1.05) | 0.285 | 1.00(0.89–1.12) | 0.984 | 0.90(0.76–1.07) | 0.220 |

Adjusted analyses using survey-weighted Cox regression were conducted for each exposure separately. Models were adjusted for age, gender, marital status, ethnicity, income, education, smoking, alcohol use, and physical activity. MetS: Metabolic syndrome, CircS: Circadian syndrome, and AL: Allostatic load, HR: Hazard ratio; CI: Confidence interval; NHANES: National Health and Nutrition Examination Surveys; MetS/CircS analytic cohort included 37304 participants, and AL analytic cohort included 26480 participants.

**Table S7. Adjusted effects of MetS, CircS, and AL on all-cause mortality using stratified Cox regression analyses, after additionally adjusting for obesity, NHANES (2005-18)**

|  | **MetS** | | **CircS** | | **AL** | |
| --- | --- | --- | --- | --- | --- | --- |
|  | **HR (95%CI)** | **p-value** | **HR (95%CI)** | **p-value** | **HR (95%CI)** | **p-value** |
| **Entire cohort** | 1.10 (1.01– 1.20) | 0.025 | 1.32 (1.21– 1.45) | <0.001 | 1.27 (1.13– 1.42) | <0.001 |
| **Age <70** | 1.30 (1.10– 1.54) | 0.002 | 1.56 (1.32– 1.84) | <0.001 | 1.45 (1.23– 1.70) | <0.001 |
| **Age ≥70** | 0.97 (0.87– 1.07) | 0.497 | 1.13 (1.02– 1.26) | 0.019 | 0.96 (0.83– 1.10) | 0.532 |

Adjusted analyses using survey-weighted stratified Cox regression were conducted for each exposure separately. Models were adjusted for age, gender, marital status, ethnicity, income, education, smoking, alcohol use, physical activity, and obesity. MetS: Metabolic syndrome, CircS: Circadian syndrome, and AL: Allostatic load, HR: Hazard ratio; CI: Confidence interval; NHANES: National Health and Nutrition Examination Surveys; MetS/CircS analytic cohort included 37304 participants, and AL analytic cohort included 26480 participants. Results from these analyses should be interpreted with caution, as an obesity-related measure, such as waist circumference, is included in the exposure definition and may lead to over-adjustment concerns.

**Table S8. Adjusted associations of MetS, CircS, and AL with all-cause mortality by age and sex, additionally adjusted with obesity using stratified Cox regression analyses, NHANES (2005-18)**

|  | **Male** |  | **Female** |  |
| --- | --- | --- | --- | --- |
|  | **HR (95%CI)** | **p-value** | **HR (95%CI)** | **p-value** |
|  | **MetS** | | | |
| **Entire cohort** | 1.09 (0.97– 1.22) | 0.138 | 1.10 (0.97– 1.26) | 0.143 |
| **Age <70** | 1.23 (0.98– 1.55) | 0.074 | 1.38 (1.04– 1.84) | 0.028 |
| **Age ≥70** | 0.97 (0.86– 1.10) | 0.675 | 0.96 (0.82– 1.12) | 0.599 |
|  | **CircS** | | | |
| **Entire cohort** | 1.39 (1.21– 1.59) | <0.001 | 1.25 (1.08– 1.44) | 0.002 |
| **Age <70** | 1.49 (1.21– 1.84) | <0.001 | 1.62 (1.25– 2.10) | <0.001 |
| **Age ≥70** | 1.24 (1.06– 1.45) | 0.007 | 1.07 (0.92– 1.25) | 0.388 |
|  | **AL** | | | |
| **Entire cohort** | 1.30 (1.06– 1.60) | 0.013 | 1.20 (1.00– 1.44) | 0.048 |
| **Age <70** | 1.53 (1.18– 1.98) | 0.002 | 1.32 (1.01– 1.72) | 0.041 |
| **Age ≥70** | 0.87 (0.71– 1.08) | 0.207 | 1.01 (0.81– 1.25) | 0.93 |

Adjusted analyses using survey-weighted stratified Cox regression were conducted for each exposure separately. Models were adjusted for age, gender, marital status, ethnicity, income, education, smoking, alcohol use, physical activity, and obesity. MetS: Metabolic syndrome, CircS: Circadian syndrome, and AL: Allostatic load, HR: Hazard ratio; CI: Confidence interval; NHANES: National Health and Nutrition Examination Surveys; MetS/CircS analytic cohort included 37304 participants, and AL analytic cohort included 26480 participants. Results from these analyses should be interpreted with caution, as an obesity-related measure, such as waist circumference, is included in the exposure definition and may lead to over-adjustment concerns.

**Table S9.** **Unadjusted and adjusted associations of MetS, CircS, and AL with all-cause mortality using logistic regression analyses, NHANES (2005-18)**

|  | **MetS** | | **CircS** | | **AL** | |
| --- | --- | --- | --- | --- | --- | --- |
|  | **OR (95%CI)** | **p-value** | **OR (95%CI)** | **p-value** | **OR (95%CI)** | **p-value** |
| **Entire cohort** |  |  |  |  |  |  |
| Unadjusted | 1.98 (1.81– 2.17) | <0.001 | 1.86 (1.67– 2.07) | <0.001 | 0.94 (0.83– 1.07) | 0.335 |
| Adjusted | 1.05 (0.95– 1.16) | 0.339 | 1.28 (1.15– 1.44) | <0.001 | 1.28 (1.10– 1.50) | 0.002 |
| **Age <70** |  |  |  |  |  |  |
| Unadjusted | 2.15 (1.84– 2.51) | <0.001 | 2.42 (2.07– 2.83) | <0.001 | 1.68 (1.42– 1.99) | <0.001 |
| Adjusted | 1.29 (1.11– 1.51) | 0.002 | 1.52 (1.30– 1.78) | <0.001 | 1.53 (1.29– 1.81) | <0.001 |
| **Age≥70** |  |  |  |  |  |  |
| Unadjusted | 0.84 (0.73– 0.96) | 0.01 | 1.08 (0.93– 1.26) | 0.325 | 0.84 (0.64– 1.10) | 0.199 |
| Adjusted | 0.91 (0.78– 1.05) | 0.189 | 1.18 (1.00– 1.38) | 0.051 | 0.86 (0.65– 1.15) | 0.313 |

Adjusted analyses using survey-weighted logistic regression were conducted for each exposure separately. Models were adjusted for age, gender, marital status, ethnicity, income, education, smoking, alcohol use, and physical activity. MetS: Metabolic syndrome, CircS: Circadian syndrome, and AL: Allostatic load, HR: Hazard ratio; CI: Confidence interval; NHANES: National Health and Nutrition Examination Surveys; MetS/CircS analytic cohort included 37304 participants, and AL analytic cohort included 26480 participants.

**Table S10.** **Unadjusted and adjusted associations of MetS, CircS, and AL with all-cause mortality by age and sex using logistic regression analyses, NHANES (2005-10 and 2015-18)**

|  | **Male** | | **Female** | |
| --- | --- | --- | --- | --- |
|  | **OR (95%CI)** | **p-value** | **OR (95%CI)** | **p-value** |
| **MetS** | | | | |
| **Entire cohort** |  |  |  |  |
| Unadjusted | 1.85 (1.65– 2.09) | <0.001 | 2.16 (1.89– 2.47) | <0.001 |
| Adjusted | 1.11 (0.96– 1.27) | 0.155 | 0.99 (0.86– 1.14) | 0.902 |
| **Age <70** |  |  |  |  |
| Unadjusted | 1.98 (1.61– 2.43) | <0.001 | 2.53 (2.02– 3.15) | <0.001 |
| Adjusted | 1.27 (1.02– 1.58) | 0.032 | 1.30 (1.02– 1.66) | 0.033 |
| **Age ≥70** |  |  |  |  |
| Unadjusted | 0.92 (0.77– 1.09) | 0.335 | 0.80 (0.66– 0.98) | 0.032 |
| Adjusted | 0.99 (0.82– 1.21) | 0.953 | 0.86 (0.70– 1.06) | 0.150 |
| **CircS** | | | | |
| **Entire cohort** |  |  |  |  |
| Unadjusted | 1.83 (1.60– 2.09) | <0.001 | 1.94 (1.67– 2.26) | <0.001 |
| Adjusted | 1.37 (1.17– 1.60) | <0.001 | 1.21 (1.02– 1.43) | 0.026 |
| **Age <70** |  |  |  |  |
| Unadjusted | 2.28 (1.89– 2.76) | <0.001 | 2.79 (2.19– 3.56) | <0.001 |
| Adjusted | 1.51 (1.24– 1.85) | <0.001 | 1.50 (1.16– 1.95) | <0.002 |
| **Age ≥70** |  |  |  |  |
| Unadjusted | 1.25 (1.00– 1.55) | 0.046 | 1.02 (0.84– 1.24) | 0.846 |
| Adjusted | 1.24 (0.97– 1.45) | 0.303 | 1.01 (0.82– 1.25) | 0.914 |
| **AL** | | | | |
| **Entire cohort** |  |  |  |  |
| Unadjusted | 0.93 (0.76– 1.12) | 0.430 | 0.94 (0.80– 1.11) | 0.475 |
| Adjusted | 1.40 (1.09– 1.79) | 0.008 | 1.15 (0.91– 1.47) | 0.244 |
| **Age <70** |  |  |  |  |
| Unadjusted | 1.59 (1.23– 2.05) | 0.001 | 1.73 (1.34– 2.24) | <0.001 |
| Adjusted | 1.64 (1.23– 2.19) | 0.001 | 1.39 (1.06– 1.81) | 0.017 |
| **Age ≥70** |  |  |  |  |
| Unadjusted | 0.86 (0.62– 1.20) | 0.366 | 0.84 (0.61– 1.17) | 0.309 |
| Adjusted | 0.82 (0.57– 1.17) | 0.266 | 0.87 (0.59– 1.30) | 0.504 |

Adjusted analyses using survey-weighted logistic regression were conducted for each exposure separately. Models were adjusted for age, gender, marital status, ethnicity, income, education, smoking, alcohol use, and physical activity. MetS: Metabolic syndrome, CircS: Circadian syndrome, and AL: Allostatic load, OR: Odds ratio; CI: Confidence interval; NHANES: National Health and Nutrition Examination Surveys; MetS/CircS analytic cohort included 37304 participants, and AL analytic cohort included 26480 participants.

**Table S11. Unadjusted and adjusted effects of MetS, CircS, and AL on all-cause mortality in sensitivity analyses restricted to complete data on all markers for computing MetS, CircS, and AL in the entire cohort and by age groups using the NHANES (2005-10 and 2015-18)**

|  | **Cox Regression** | | **Logistic Regression** | |
| --- | --- | --- | --- | --- |
|  | **HR (95%CI)** | **p-value** | **OR (95%CI)** | **p-value** |
| **MetS** | | | | |
| **Entire cohort** |  |  |  |  |
| Unadjusted | 2.07(1.79–2.39) | <0.001 | 2.23(1.93–2.59) | <0.001 |
| Adjusted | 1.15(1.00–1.31) | 0.044 | 1.11(0.95–1.29) | 0.178 |
| **Age <70** |  |  |  |  |
| Unadjusted | 2.20(1.76–2.75) | <0.001 | 2.30(1.84–2.88) | <0.001 |
| Adjusted | 1.37(1.11–1.68) | 0.003 | 1.32(1.07–1.62) | 0.011 |
| **Age ≥70** |  |  |  |  |
| Unadjusted | 0.96(0.81–1.13) | 0.607 | 0.99(0.81–1.2) | 0.888 |
| Adjusted | NA |  | NA |  |
| **CircS** | | | | |
| **Entire cohort** |  |  |  |  |
| Unadjusted | 2.03(1.74–2.38) | <0.001 | 2.1(1.79–2.47) | <0.001 |
| Adjusted | 1.31(1.13–1.51) | <0.001 | 1.3(1.09–1.55) | 0.003 |
| **Age <70** |  |  |  |  |
| Unadjusted | 2.52(2.02–3.16) | <0.001 | 2.55(2.03–3.2) | <0.001 |
| Adjusted | 1.63(1.31–2.04) | <0.001 | 1.55(1.23–1.96) | <0.001 |
| **Age ≥70** |  |  |  |  |
| Unadjusted | 1.09(0.93–1.26) | 0.281 | 1.13(0.91–1.4) | 0.26 |
| **AL** | | | | |
| **Entire cohort** |  |  |  |  |
| Unadjusted | 1.07(0.95–1.21) | 0.235 | 1.03(0.92–1.16) | 0.596 |
| Adjusted | 1.31(1.16–1.48) | <0.001 | 1.34(1.15–1.57) | <0.001 |
| **Age <70** |  |  |  |  |
| Unadjusted | 1.65(1.37–1.99) | <0.001 | 1.81(1.53–2.14) | <0.001 |
| Adjusted | 1.51(1.26–1.81) | <0.001 | 1.63(1.37–1.95) | <0.001 |
| **Age ≥70** |  |  |  |  |
| Unadjusted | 0.91(0.77–1.07) | 0.255 | 0.88(0.68–1.14) | 0.317 |
| Adjusted | 0.95(0.82–1.1) | 0.504 | 0.87(0.66–1.14) | 0.309 |

Adjusted analyses using survey-weighted stratified Cox regression and logistic regression were conducted for each exposure separately. Models were adjusted for age, gender, marital status, ethnicity, income, education, smoking, alcohol use, and physical activity. MetS: Metabolic syndrome, CircS: Circadian syndrome, and AL: Allostatic load, HR: Hazard ratio; OR: Odds ratio; CI: Confidence interval; NHANES: National Health and Nutrition Examination Surveys; Analytic cohort included 26328 participants.

**Table S12. Unadjusted and adjusted effects of MetS, CircS, and AL on all-cause mortality by sex in sensitivity analyses restricted to complete data on all markers for computing MetS, CircS, and AL using the NHANES (2005-10 and 2015-18)**

|  | **Cox Regression** | | **Logistic Regression** | |
| --- | --- | --- | --- | --- |
|  | **HR (95%CI)** | **p-value** | **OR (95%CI)** | **p-value** |
| **MetS** | | | | |
| **Male** |  |  |  |  |
| Unadjusted | 1.86(1.55–2.24) | <0.0001 | 1.98(1.63–2.42) | <0.0001 |
| Adjusted | 1.16(0.96–1.40) | 0.121 | 1.10(0.89–1.37) | 0.355 |
| **Female** |  |  |  |  |
| Unadjusted | 2.36(1.85–3.01) | <0.0001 | 2.58(2.04–3.27) | <0.0001 |
| Adjusted | 1.13(0.90–1.42) | 0.292 | 1.10(0.85–1.42) | 0.478 |
| **CircS** | | | | |
| **Male** |  |  |  |  |
| Unadjusted | 2.05(1.73–2.43) | <0.0001 | 2.10(1.74–2.53) | <0.0001 |
| Adjusted | 1.43(1.18–1.73) | <0.0001 | 1.44(1.15–1.81) | 0.002 |
| **Female** |  |  |  |  |
| Unadjusted | 2.05(1.59–2.65) | <0.0001 | 2.14(1.65–2.77) | <0.0001 |
| Adjusted | 1.16(0.93–1.44) | 0.19 | 1.13(0.86–1.49) | 0.369 |
| **AL** | | | | |
| **Male** |  |  |  |  |
| Unadjusted | 1.06(0.90–1.25) | 0.469 | 1.00(0.83–1.21) | 0.976 |
| Adjusted | 1.38(1.14–1.68) | 0.001 | 1.44(1.12–1.87) | 0.006 |
| **Female** |  |  |  |  |
| Unadjusted | 1.07(0.90–1.28) | 0.432 | 1.05(0.87–1.26) | 0.628 |
| Adjusted | 1.21(1.00–1.45) | 0.045 | 1.22(0.94–1.57) | 0.133 |

Adjusted analyses using survey-weighted stratified Cox regression and logistic regression were conducted for each exposure separately. Models were adjusted for age, gender, marital status, ethnicity, income, education, smoking, alcohol use, and physical activity. MetS: Metabolic syndrome, CircS: Circadian syndrome, and AL: Allostatic load, HR: Hazard ratio; OR: Odds ratio; CI: Confidence interval; NHANES: National Health and Nutrition Examination Surveys; Analytic cohort included 26328 participants.

**Table S13. Adjusted effect of composite exposure variable on all-cause mortality in sensitivity analyses restricted to complete data on all markers for computing MetS, CircS, and AL in the entire cohort and by age groups using the NHANES (2005-10 and 2015-18)**

|  | **Cox Regression** | | **Logistic Regression** | |
| --- | --- | --- | --- | --- |
|  | **HR (95%CI)** | **p-value** | **OR (95%CI)** | **p-value** |
| **Entire cohort** |  |  |  |  |
| No MetS/CircS/AL (reference) |  |  |  |  |
| MetS only | 0.94(0.81–1.10) | 0.440 | 0.83(0.71–0.97) | 0.020 |
| Primary CircS | 1.23(1.08–1.39) | 0.002 | 1.25(1.06–1.47) | 0.008 |
| Primary AL | 1.06(0.88–1.27) | 0.561 | 1.05(0.85–1.29) | 0.631 |
| **Age<70** |  |  |  |  |
| No MetS/CircS/AL (reference) |  |  |  |  |
| MetS only | 1.07(0.79–1.43) | 0.663 | 1.00(0.72–1.37) | 0.987 |
| Primary CircS | 1.39(1.09–1.77) | 0.009 | 1.49(1.18–1.88) | 0.001 |
| Primary AL | 1.25(0.97–1.60) | 0.080 | 1.33(1.03–1.71) | 0.028 |
| **Age≥70** |  |  |  |  |
| No MetS/CircS/AL (reference) |  |  |  |  |
| MetS only | 0.84(0.72–0.98) | 0.023 | 0.72(0.58–0.89) | 0.002 |
| Primary CircS | 1.06(0.95–1.17) | 0.303 | 1.14(0.92–1.40) | 0.222 |
| Primary AL | 0.83(0.65–1.06) | 0.136 | 0.75(0.54–1.04) | 0.083 |

Adjusted analyses using survey-weighted stratified Cox and logistic regressions were conducted for combined exposure. Models were adjusted for age, gender, marital status, ethnicity, income, education, smoking, alcohol use, and physical activity. MetS: Metabolic syndrome, CircS: Circadian syndrome, and AL: Allostatic load, HR: Hazard ratio; OR: Odds ratio; CI: Confidence interval; NHANES: National Health and Nutrition Examination Surveys; Analytic cohort included 26328 participants. Results may be biased by a combined exposure definition and should be interpreted with caution.

**Table S14. Simultaneous adjusted effects of all exposure variables on all-cause mortality after ignoring overlapping across exposures in sensitivity analyses restricted to complete data on all markers for computing MetS, CircS, and AL in the entire cohort and by age groups using the NHANES (2005-10 and 2015-18)**

|  | **Cox Regression** | | **Logistic Regression** | |
| --- | --- | --- | --- | --- |
| **Entire cohort** | **HR (95%CI)** | **p-value** | **OR (95%CI)** | **p-value** |
| **MetS** | 0.95(0.83–1.09) | 0.443 | 0.77(0.67–0.88) | <0.001 |
| **CircS** | 1.25(1.08–1.44) | 0.003 | 1.49(1.27–1.75) | <0.001 |
| **AL** | 1.20(1.09–1.34) | 0.001 | 1.25(1.08–1.45) | 0.003 |
| **Age <70** |  |  |  |  |
| **MetS** | 0.85(0.67–1.09) | 0.204 | 0.85(0.66–1.10) | 0.22 |
| **CircS** | 1.37(1.05–1.78) | 0.019 | 1.46(1.13–1.88) | 0.004 |
| **AL** | 1.38(1.18–1.62) | <0.001 | 1.45(1.23–1.71) | <0.001 |
| **Age ≥70** |  |  |  |  |
| **MetS** | 0.93(0.81–1.08) | 0.336 | 0.71(0.58–0.87) | 0.001 |
| **CircS** | 1.18(1.02–1.37) | 0.029 | 1.60(1.29–1.99) | <0.001 |
| **AL** | 0.94(0.81–1.08) | 0.343 | 0.84(0.63–1.13) | 0.249 |

Adjusted analyses using survey-weighted stratified Cox and logistic regressions were conducted for all exposures in a single model, ignoring construct overlap. Models were adjusted for age, gender, marital status, ethnicity, income, education, smoking, alcohol use, and physical activity. MetS: Metabolic syndrome, CircS: Circadian syndrome, and AL: Allostatic load, HR: Hazard ratio; OR: Odds ratio; CI: Confidence interval; NHANES: National Health and Nutrition Examination Surveys; Analytic cohort included 26328 participants. Results may be biased by collinearity and should be interpreted with caution.

**Table S15. Performance metric (Harrell’s c-statistic) for Cox models with each exposure**

| **Cohort** | **Models** | **MetS** | **CircS** | **AL** |
| --- | --- | --- | --- | --- |
| **Entire cohort** | Unadjusted | 0.57 (0.56- 0.58) | 0.55 (0.54- 0.56) | 0.51 (0.5- 0.52) |
|  | Adjusted | 0.86 (0.86- 0.87) | 0.86 (0.86- 0.87) | 0.87 (0.86- 0.87) |
| **Age<70** | Unadjusted | 0.57 (0.56- 0.58) | 0.55 (0.54- 0.56) | 0.49 (0.48- 0.5) |
|  | Adjusted | 0.85 (0.85- 0.86) | 0.85 (0.85- 0.86) | 0.86 (0.86- 0.87) |
| **Age ≥70** | Unadjusted | 0.43 (0.42- 0.44) | 0.55 (0.54- 0.56) | 0.51 (0.5- 0.52) |
|  | Adjusted | 0.86 (0.85- 0.86) | 0.86 (0.85- 0.86) | 0.86 (0.86- 0.87) |

Adjusted analyses using survey-weighted stratified Cox regression were conducted for each exposure separately. Models were adjusted for age, gender, marital status, ethnicity, income, education, smoking, alcohol use, and physical activity. MetS: Metabolic syndrome, CircS: Circadian syndrome, and AL: Allostatic load, CI: Confidence interval; NHANES: National Health and Nutrition Examination Surveys; MetS/CircS analytic cohort included 37304 participants, and AL analytic cohort included 26480 participants.

**Table S16. Incremental differences in prediction accuracies by inclusion of each exposure in adjusted analysis compared to a base model including only baseline covariates in the entire cohort and by age groups using the NHANES (2005-18)**

|  | **Base +exposure model** | **Base model** | **Difference** |
| --- | --- | --- | --- |
|  | **C-statistic(95%CI)** | **C-statistic(95%CI)** |  |
| **MetS** |  |  |  |
| **Entire cohort** | 0.86(0.87–0.86) | 0.86(0.86–0.87) | 0.000 |
| **Age<70** | 0.85(0.86–0.86) | 0.86(0.85–0.86) | -0.001 |
| **Age≥70** | 0.86 (0.85-0.86) | 0.68(0.67–0.69) | 0.174 |
| **CircS** |  |  |  |
| **Entire cohort** | 0.86(0.87–0.86) | 0.86(0.86–0.87) | 0.002 |
| **Age<70** | 0.85(0.86–0.86) | 0.86(0.85–0.86) | -0.001 |
| **Age≥70** | 0.85(0.86–0.68) | 0.68(0.67–0.69) | 0.174 |
| **AL** |  |  |  |
| **Entire cohort** | 0.86(0.87–0.87) | 0.87(0.86–0.87) | 0.001 |
| **Age<70** | 0.86(0.87–0.86) | 0.86(0.86–0.87) | 0.000 |
| **Age≥70** | 0.86(0.87–0.68) | 0.68(0.67–0.69) | 0.180 |

Adjusted models were conducted using survey-weighted stratified Cox regression. Base plus exposure models included exposure along with age, gender, marital status, ethnicity, income, education, smoking, alcohol use, and physical activity. Base models included all baseline covariates except for exposure. MetS: Metabolic syndrome, CircS: Circadian syndrome, and AL: Allostatic load, HR: Hazard ratio; CI: Confidence interval; NHANES: National Health and Nutrition Examination Surveys; Analytic cohort included 26328 participants.

**Table S17. Prognostic score adjusted effects of MetS, CircS, and AL on all-cause mortality in the entire cohort and individuals aged<70 years, using stratified Cox regression analyses, NHANES (2005-18)**

|  | **HR (95%CI)** | **p-value** | **C-statistic (95%CI)** |
| --- | --- | --- | --- |
| **Entire cohort** |  |  |  |
| **MetS** | 1.26(0.94–1.70) | 0.126 | 0.53(0.49–0.56) |
| **CircS** | 1.47(1.08–2.00) | 0.015 | 0.55(0.51–0.59) |
| **AL** | 1.57(1.15–2.15) | 0.005 | 0.55(0.51–0.58) |
| **Age<70** |  |  |  |
| **MetS** | 1.43(1.07–1.91) | 0.017 | 0.54(0.51–0.57) |
| **CircS** | 1.62(1.19–2.20) | 0.003 | 0.55(0.52–0.58) |
| **AL** | 1.62(1.19–2.20) | 0.003 | 0.54(0.51–0.57) |

Prognostic adjusted analyses using survey-weighted stratified Cox regression were conducted for each exposure separately. Prognostic models included exposure, age, gender, marital status, ethnicity, income, education, smoking, alcohol use, and physical activity. MetS: Metabolic syndrome, CircS: Circadian syndrome, and AL: Allostatic load, HR: Hazard ratio; CI: Confidence interval; NHANES: National Health and Nutrition Examination Surveys; MetS/CircS analytic cohort included 37304 participants, and AL analytic cohort included 26480 participants.
